# Where scrollbars are clicked, and why

**DOI:** 10.1186/s41235-024-00551-z

**Published:** 2024-04-19

**Authors:** Oliver Herbort, Philipp Raßbach, Wilfried Kunde

**Affiliations:** Department of Psychology, University of Würzburg, Röntgenring 11, 97070 Würzburg, Germany

**Keywords:** Scrolling, Anticipation, Action planning, Human–computer interaction

## Abstract

Scrolling is a widely used mean to interact with visual displays, usually to move content to a certain target location on the display. Understanding how user scroll might identify potentially suboptimal use and allows to infer users’ intentions. In the present study, we examined where users click on a scrollbar depending on the intended scrolling action. In two online experiments, click positions were systematically adapted to the intended scrolling action. Click position selection could not be explained as strict optimization of the distance traveled with the cursor, memory load, or motor-cognitive factors. By contrast, for identical scrolling actions click positions strongly depended on the context and on previous scrolls. The behavior of our participants closely resembled behavior observed for manipulation of other physical devices and suggested a simple heuristic of movement planning. The results have implications for modeling human–computer interaction and may contribute to predicting user behavior.

**Supplementary Information:**

The online version contains supplementary material available at 10.1186/s41235-024-00551-z.

## Public Significance Statement

Typical computer applications, such as maps, websites, or text documents, involve some form of scrolling. Scrolling requires linear cursor or finger movements, whose maximal extent is limited by screen borders and other constraints. We show that participants adapt their clicks on scrollable objects to the intended scrolling actions, thus anticipating constraints on possible movements and reducing their impact. Our findings could add an important aspect to models of human–computer interaction and offers one avenue for predicting user behavior.

## Introduction

Most of us interact with desktop computers, tablets, or cell phones every day. Due to limited screen or window sizes, the information that we seek often needs to be brought to the display first. This is often accomplished with scrolling. We scroll through lists, websites, or text files, but also scroll to adjust the view of maps or in 3D applications. Average computer users have been estimated to make about 600 mouse clicks per hour (Taylor, 2007). Even if only a fraction of these clicks involves scrolling, it is plainly obvious that scrolling is a very prevalent behavior of humans nowadays, when taking worldwide computer use into account. It is thus no wonder that extensive research has been devoted to understanding this kind of user interaction. For example, Fitts’ Law (Fitts, 1954), which describes the relationship between the duration, amplitude, and target size of goal-directed movements, has been applied to scrolling (e.g., Zhao et al., 2014, 2015). Likewise, the efficiency of different input devices and modes of scrolling has been examined (e.g., Chen & Proctor, 2013; Zhai et al., 1997; Zhao et al., 2014).

Scrolling with the mouse cursor typically requires one or more continuous, linear mouse movements while pressing a button. Scrolling with a scroll wheel, touch screen, or by using gestures on a track pad is usually realized by continuous linear movements of one or more fingers. For example, scrolling through a list may be realized by clicking on the list with the cursor and then moving the cursor linearly up or down, or by vertical linear finger movements on a touch screen. In all cases, the maximal amplitude of the linear movements is limited. Cursor movements are limited by screen or window borders and properties of the physical workspace, such as the edge of a mouse pad. Likewise, the maximal amplitude of finger movements is limited by the boundaries of screens, trackpads, or scroll wheels. Although some systems bend these boundaries by allowing interactions with offscreen objects (Markussen et al., 2016; Takashima et al., 2015), most human–computer interactions are subjected to these limitations. Hence, repeated cursor or finger movements are often necessary to accomplish an intended scroll.

Despite the limits dictated by screens or input devices, users have some influence on how far the cursor or finger can be moved in a single stroke by selecting a start point for the cursor or fingers. For example, when scrolling to the right with a touch screen, the finger is typically placed on the left part of the screen and, vice versa, placed on the right when scrolling to the left (Zhao et al., 2014). How exactly participants select such start positions for scrolling actions has received little scientific attention so far. Hence, the goal of this articles is to examine how the location at which a scrollable object is clicked depends on the desired amplitude of the object movement. First, examining this relationship might allow a better understanding of how we plan action sequences. Second, a regular relationship between click position and the magnitude of the upcoming scrolling action may be an important aspect for modeling user behavior, may be a useful and no-cost cue to predict upcoming actions, and thus improve human–computer interactions. Third, albeit we report data on a scrolling task, we expect that our conclusions also apply to tasks that are subject to similar constraints, such as drag-and-drop actions (e.g., moving files on the desktop or moving a textbox in a presentation file).

Albeit click position selection in anticipation of scrolling actions has received little attention, analogous behavior has been extensively studied in the domain of physical object manipulations (Rosenbaum et al., 2012). For example, when participants were asked to grasp and rotate a dial, the orientation of the hand when grasping the dial was inversely related to the direction and magnitude of the intended dial rotation (Herbort & Butz, 2010). This effect not only applies to the manipulation of real objects but also to virtual rotary controls (Olafsdottir et al., 2014). Likewise, when participants were asked to move a vertically oriented rod to a higher or lower position, the grasp positions were inversely related to the upcoming rod displacement (Cohen & Rosenbaum, 2004). In both examples, participants needed to continuously move the hand to realize the desired object manipulation while biomechanical constraints, such as the maximum range of joint angles, limited the potential extent of these movements. However, by adjusting the grasp, the potential extent of continuous rotary or linear hand movements was increased. Moreover, participants selected grasp positions that facilitated the upcoming movement, for example, by increasing the control over the manipulated object before placing it (Herbort & Kunde, 2019; Rosenbaum et al., 1996). In summary, grasps for object manipulation are finely tuned to the direction and extent of the subsequent object manipulation and reduce the impact of the limits of the human motor system. That is, a first action (i.e., grasping) is planned with respect to the requirements of a subsequent action (i.e., object manipulation). This mode of planning is called “second-order planning” (Rosenbaum et al., 2012). Participants face a conceptually similar problem during scrolling, with the difference that limitations do not result from biomechanics but from the work environment or the user interface (edges of mouse pads, touch pads, or screens). Here, second-order planning such as adapting the click position of a cursor or the finger placement on a touch pad, for example, might increase the range of scrolling actions that can be completed in one go.

## Experiment 1

**Fig. 1:**
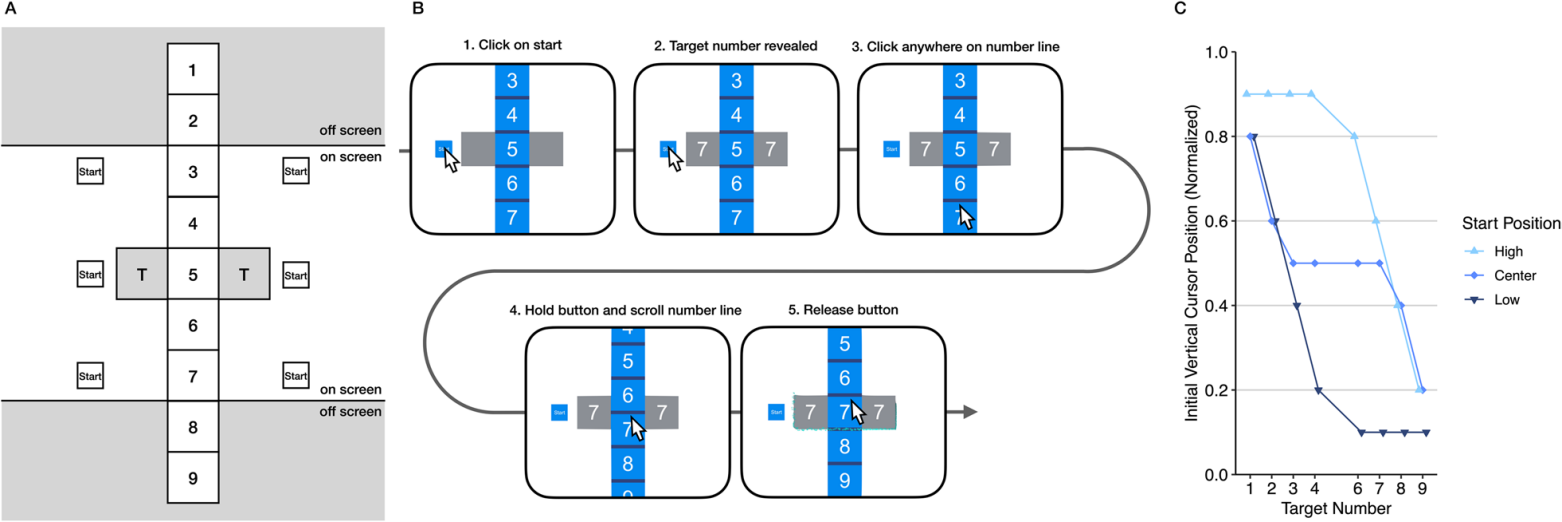
Stimuli, trial procedure, and predictions for Experiment 1. Note: **A** The chart shows the elements of the scrolling task of Experiment 1. Of the six possible starting position only one was displayed on each individual trial. Not the entire number line was visible at trial onset. **B** The panel shows the sequence of events in a trial. If the target number was centered between the target squares in step 5, the trial ended. Otherwise, participants could re-click the number line (step 3). **C** The chart shows the predicted results if participants minimize overall movement distance.

Experiment 1 was conducted to test whether participants rely on second-order planning when selecting the initial cursor position for scrolling and how exactly the initial finger position is determined based on the expected direction and magnitude of the scrolling movement. In Experiment 1, participants moved the mouse cursor from a predefined starting position to a number line, clicked on the number line, and then moved the number line up or down by a prespecified amplitude (Fig. 1A, B). Participants were allowed to complete the scrolling action by repeatedly releasing and re-clicking the number line.

As the joint-angle-based planning criteria that are involved in the physical tasks described above are not a major constraint in typical scrolling actions, we assumed that participants select click positions that minimize the distance traveled with the mouse cursor (and in our case thus the overall movement time) and that the extent of mouse movements is only limited by the borders of the screen. The precise predictions of this model are shown in Fig. 1C and will be explained in detail at the end of the method section.

### Methods

#### Participants

Complete datasets of 36 participant were collected in 2021 (19 female, 17 male; 28 right handed, 7 left handed, 1 ambidextrous). Their mean age was 26 years and ranged from 20 to 63 years. They were recruited from the participant pool of the Department of Psychology of the University of Würzburg and received 5€. Participants resided predominantly in Würzburg or other German cities. Participants were instructed to use the mouse. Whereas most participants reported to have used the mouse (n = 17), others used the touchpad (n = 16), and three used both input devices. As behavior did not differ qualitatively between groups, we included all participants in the statistical analyses.

For the power analysis, we simulated experiments in which participants behaved according to predictions but with normal distributed noise added to the data points of each participant (but with click positions constrained to the screen). Thus, data points of individual participants were uncorrelated. For simulations, we used noise SDs of 20% and 40% of screen height. The actual between-participant SDs of the different conditions were on average 7% of the screen height, for comparison. We simulated 10.000 experiments with sample sizes of 18, 24, 30, and 36 participants. For the smaller SD, the simulated powers (1-β) for the main effects of start position and target number as well as the interactions were 100% for all tested sample sizes. For the larger SD, power exceeded 95% for all effects for sample sizes of 24 participants upward. To be on the safe side, we collected data of 36 participants.

### Stimulus and apparatus

The experiment was conducted online with a custom-build application for PC and Mac, which was developed with the Unity engine (Unity Technologies, 2019). A screen recording of exemplar trials is provided as supplement. All stimuli were scaled to the height of the participants’ screens. In the following, dimensions are given relative to screen height (= 1 unit). Figure 1A shows the elements involved in each trial. A vertical number line was centered on the screen. The number line consisted of nine blue squares (edge length 0.2 units) containing white numbers from one to nine from top to bottom. Note that only a fraction of the number line was visible at each moment of the experiment. Two gray target squares (edge length: 0.2 units) were centered vertically and placed 0.2 units to the left and right of the screen center. In addition, a start square (edge length 0.1 units) was displayed 0.4 units to the left or right of the screen center and either vertically centered or 0.4 units above or below the screen center.

### Procedure

Participants downloaded and started the application. After being prompted for age, handedness, and gender, instructions were presented. After that, the experiment started. Each trial of the experiment started with the presentation of the centered number line (Fig. 1B) with the numbers from three to seven being visible on screen, the two empty target squares, and the start square. Participants were instructed to first click on the start square with the left mouse button (from here on: button). Once participants released the button while on the start square, the target numbers were shown within the target squares. Participants were instructed to drag the number line so that the target number on the number line aligned with the target numbers in the target squares. When participants pressed and held the button on any position on the number line, the number line moved vertically in synchrony with the y-coordinate of the cursor. When participants released the button, the number line remained in its last position irrespective of further cursor movements. Participants could then click on any position on the number line to move it again. If participants released the number line with the target number within 0.02 units of the screen center, the number line aligned itself with the target squares and a short animation was played for one second (a blue spark encircled the target squares and the number line square between them). The next trial started directly after the animation. At the end of the experiment, a short questionnaire was administered, and the data were either automatically uploaded to a server or participants emailed their data.

The experiment consisted of eight blocks. Each combination of the factors target number (1, 2, 3, 4, 6, 7, 8, 9), vertical start position (low, center, high), and horizontal start position (left, right) was presented once in a block. Trial order was randomized. A self-paced break was scheduled every two blocks.

### Data reduction and analysis

During the experiment, we collected the positions of the cursor and the number line, whenever participants clicked or released the mouse button. The following variables were analyzed. The central dependent variable was the *initial vertical cursor position* (or *click position* for short), which was the vertical position of the cursor when participants first clicked on the number line. The cursor coordinates were normalized with respect to participants screen heights, so that 0.0 corresponded to the bottom edge of the screen and 1.0 corresponded to the top edge. In addition, we extracted the following variables. Albeit these variables are not central for our hypotheses, we included them to provide a more complete picture of participants’ behavior. The *final vertical cursor position* was defined as the vertical cursor position at the end of the last scroll. The *number of submovements* was defined as the number of times the number line was clicked and released. The *response time* was defined as the time between target number onset and end of the final submovement. Sixty-three trials (0.5%) were excluded because the time from start square onset until trial completion exceeded 10 s. All other trials were used for analysis. Data analysis was conducted with R (R Core Team, 2022) and the *afex* package (Singmann et al., 2023).

### Predictions

We predicted that participants select click positions that result in the shortest overall cursor trajectory. In the context of our experiments, we focus on the prediction of the vertical component of the click position. The selection of the click positions affects the overall trajectory length in two ways. First, the click position has a direct effect on the length of the initial movement from the start position to the clicked position on the number line. Second, the click position determines whether the number line can be moved in one go. If this is not the case, that is, when the cursor reaches the screen border before the number line can be centered, participants must release the number line and travel back with the cursor before they continue the scrolling action. Traveling back with the cursor further increases the trajectory length. To predict click positions, we computed the click positions that resulted in the shortest overall cursor trajectory length for each condition, assuming linear trajectories and no unnecessary movements like moving the scroll bar back and forth. The code is provided in the file power_analysis_exp_1.R in the supplemental material. The predictions of this simple model are outlined in Fig. 1C, which lets us expect a considerable effect of the start position, the target number, and an interaction between both.

## Results

The horizontal start position was not included as a factor, because we were mostly interested in the vertical component of the task and because the factor had no effect (Table ESM-1). The input device had a numerically small but significant effect on initial vertical cursor positions. Generally, click positions of touchpad users tended to be shifted toward the screen center by about 7% when compared to mouse users (Table ESM-2, Fig. ESM-1). We did not include this factor due to the descriptively similar results and small sub-group sizes but return to potential influences of the input device in the general discussion.

**Fig. 2:**
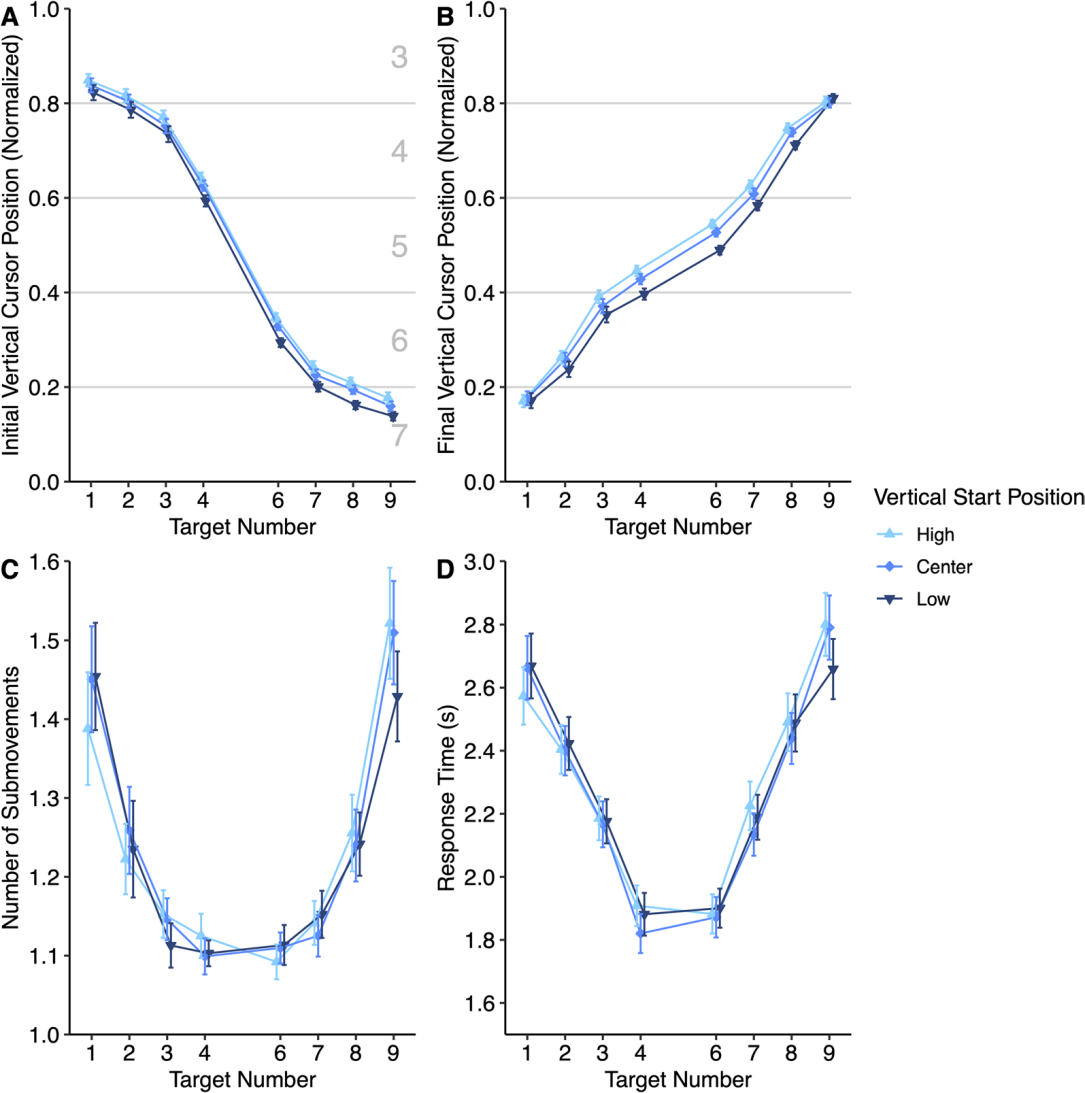
Results of Experiment 1. Note: A, B). The figures show the effect of target number and start position on the initial (**A**) and final (**B**) vertical cursor position. Values of 0 and 1 refer to the bottom and top edge of the screen, respectively. The gray lines and numbers indicate the positions of the initially visible number line squares for reference. **C**, **D** The figures show the effect of target number and start position on the number of submovements (**C**) and response times (**D**). Error bars show 1 standard error of the mean

The dependent variables were subjected to within-participants analyses of variance (ANOVA) with factors vertical start position and target number, applying the Greenhouse–Geisser correction for sphericity violations. Figure 2A shows the initial vertical cursor position. The vertical cursor position of the first click on the number line was inversely related to the upcoming scrolling actions, *F*(1.48,51.92) = 699.17, *p* < .001, η^2^_*G*_ = .94, ε = .21. That is, downward scrolls resulted in high click positions and upward scrolls in low click positions. Consecutive contrasts revealed that click positions differed for all adjacent pairs of target numbers, all *t*(35)s ≥ 5.32, *p*s ≤ .001, *d*_*z*_s ≥ .89. In addition, initial vertical cursor positions were biased toward the position of the start square, *F*(1.29,44.99) = 51.05,* p* < .001, η^2^_*G*_ = *.0*5, ε = .64. Consecutive contrasts revealed a difference between the low and center start position, *t*(35) = 8.10, *p* < . 001, *d*_*z*_ = 1.35, and the center and high start position, *t*(35) = 4.73, *p* < .001, *d*_*z*_ = 0.79. Both factors did not interact, *F*(8.26,289.24) = 1.59, *p* = .126, η^2^_*G*_ = *.0*0, ε = .59.

Figure 2B shows that the final vertical cursor position depended on the target number, *F*(2.67,93.31) = 501.71, *p* < .001, η^2^_*G*_ = 0.91, ε = 0.38. Likewise, the start position affected the final cursor position, *F*(1.50,52.49) = 43.47, *p* < .001, η^2^_*G*_ = *.0*3, ε = 0.75. Both factors interacted, *F*(9.18,321.35) = 7.27, *p* < .001, η^2^_*G*_ = *.0*2, ε = .66. Descriptively, the effect of the start position vanished for the furthest scrolling actions.

Figure 2C shows the number of submovements. The higher the amplitude of the scrolling action, the higher was the number of submovements, *F*(1.73,60.55) = 27.77, *p* < .001, η^2^_*G*_ = 0.21, ε = .25. Furthermore, target number and start position interacted, *F*(7.83,274.15) = 2.38, *p* = .018, η^2^_*G*_ = .01, ε = .56. Descriptively, more submovements were required for far downward scrolls (target numbers 1 and 2) when starting low and far upward scrolls (target numbers 8 and 9) when starting high than for the reverse conditions. The start position did not have a significant effect, *F*(1.96,68.45) = 0.89, *p* = *.4*13, η^2^_*G*_ = *.0*0, ε = .98. Submovements were either executed to correct for an inaccurately placed number line at the end of the movement (55%, operationalized as submovements requiring less than 0.1 units to allow perfect alignment of the number line) or to cover a more substantial part of the required scrolling movement (45%). A few submovements (7% of the latter) did not involve a substantial repositioning of the cursor (less than 0.1 units) and apparently resulted from releasing and re-clicking the number line.

Finally, the response times followed the pattern of the number of submovements, revealing a main effect of target number, *F*(2.83,99.10) = 170.75, *p* < .001, η^2^_*G*_ = *.2*9, ε = .40, and the interaction, *F*(8.18,286.30) = 3.35, *p* = .001, η^2^_*G*_ = .00, ε = .58. The interaction reflects the different amplitudes of the movements from the start square to the number line. Again, the start position did not have a significant effect, *F*(1.92,67.36) = 1.64, *p* = *.2*03, η^2^_*G*_ = .00, ε = .96.

### Short discussion

Experiment 1 addressed whether and how participants adapt click positions to upcoming scrolling actions. The experiment revealed a clear relationship between click positions and the direction of the upcoming scrolling action. In addition, the start position had a small effect on click positions. This effect fully carried over to the final cursor positions for shorter scrolls, as they were typically completed in one go. By contrast longer scrolls required additional submovements for all but the most excursed initial click positions (Fig. 2C), which systematically washed out any differences in initial click positions.

We expected that participants clicked on the closest position on the number line that would allow moving the number line in one go thus minimizing the overall length of the cursor trajectory. The data fully disprove this hypothesis, as revealed by the lack of an interaction between start position and target number on initial vertical cursor positions. In addition, we predicted that target number affects click positions only slightly for more central target (e.g., 4 vs. 6) numbers and much stronger for extreme target numbers (e.g., 1 vs. 3) but found the reverse pattern. Finally, to illustrate that click positions cannot be well described by assuming that overall trajectory length is minimized, consider target number 3. This number could be centered with a single scrolling action by clicking anywhere on the numbers 3, 4, or 5. If participants started next to square 3 (high), they clicked on a more distant square than necessary in 52% of trials. Likewise, when participants started low or in the middle, a more distant square than necessary (i.e., more distant than square 5) was clicked in 86% of trials. A similar pattern was found for target numbers 2 to 8,^1^ in which further than necessary initial movements were made in 13% to 54% of those trials in which the click position allowed for centering the target in one go. In summary, participants frequently selected click positions that required a longer movement from start to number line than necessary. Moreover, also an inspection of the individual datasets revealed no individual participants conforming to our original hypotheses (Figure ESM-2). Hence, we suggest that minimizing the distance traveled with the cursor is not a major constraint in click position selections.

Alternatively, one might suggest that participants tend to click on the number line square that contains the target number, if possible, and the closest number to it otherwise. This might be done to offload memory for the target number as participants would not have to remember the target number any longer once they had placed the cursor there (Fournier et al., 2019; Risko & Gilbert, 2016). Alternatively, participants might benefit from reduced motor-cognitive costs caused by offsets between the cursor and the relevant object part (c.f., Paljic et al., 2002). However, although this hypothesis might explain why the start position only had a small effect, it can only offer a partial explanation of the data. On the one hand, inspection of individual datasets revealed that at least some participants might have adhered to this hypothesis (e.g., 6 out of 36 participants clicked on the target number on the number line in at least 80% of trials). On the other hand, participants appeared to follow this strategy in only about 60% of trials on average. Moreover, participants often did not click on the target number on the number line although it was the closest number to their starting position (e.g., when the target number was 3 and the start position was high; see previous paragraph).

Another alternative might be that participants tried to exploit that the mouse cursor cannot travel beyond the screen borders. That is, if they manage to click on the appropriate area on the number line, they could complete the scroll by moving the cursor rapidly to the edge of the screen without caring about movement accuracy. However, while this approach could also explain the minimal effect of the start position, it would predict that final cursor positions would be frequently at the edge of the screen. Figure 2B shows that this was clearly not the case. Likewise, no individual participant appeared to have used this strategy.

Finally, it is noteworthy that the data pattern closely resembled how participants rotate their hand when grasping a dial—such as the volume control of a stereo—for rotation (Herbort, 2013; Herbort & Butz, 2010; Olafsdottir et al., 2014). Grasp selections could be modeled by assuming that participants select grasps in a two-component process (Herbort & Butz, 2012). First, a prone (inward rotated) grasp or supine (outward rotated) grasp posture is selected based on the rotation direction. Second, this grasp posture is then further adapted based on the amplitude of the required object rotation, resulting in more medial grasp postures for short rotations and more excursed arm postures for far rotations. Applied to the current experiment, one could hypothesize that participants likewise use a two-component process. First, they selected a click position exclusively on the scrolling direction. The position would fall somewhere on the upper half of the number line for downward scrolls and the lower half of the number line for upward scrolls. The exact position is expected to depend on the typical requirements of the task, being closer to the number line center when shorter scrolls are frequently required and closer to its endpoints when further scrolls are frequently required. In the following, we refer to the set of scrolling actions that has been experienced in recent trials as task context. Second, this click position is then shifted inward or outward depending on the amplitude of the upcoming scrolling action. Importantly, this amplitude-based adjustment does not fully reflect the amplitude of the upcoming scroll. That is, increasing the amplitude of a scroll by 100 pixels while maintaining its direction might only result in a shift of the click position by, for example, 50 pixels. Such a model could explain the considerable effect of the scroll direction per se, as well as the consistent but comparatively small effect of the amplitude of the planned scroll on the click position. However, while this account would be in line with the present results, it is also much less constrained than the other accounts and might be fitted post hoc to various data patterns. Hence, we conducted Experiment 2 to follow up on this hypothesis.

## Experiment 2

Experiment 1 showed that participants adapt click positions to upcoming scrolling actions. However, optimality criteria such as the minimization of overall cursor path length, the reduction of motor-cognitive demands, or the exploitation of the screen border as a buffer stop, could not explain the data. By contrast, the data are in line with the assumption that participants determine click position in a two-component process, in which the click position is primarily selected based on the scroll direction and further adjusted to the amplitude of the scroll. According to this model, the click position component that is based on movement direction depends on typical task requirements. For example, positions might be selected that are suitable to accomplish most upward or downward scrolls in one go. Thus, when participants are often required to scroll the number line by a small amplitude, click positions are expected to be generally closer to its center. By contrast, when participants often execute high-amplitude scrolls, click positions are expected to be closer to its endpoints. Experiment 2 was designed to test this hypothesis. Participants worked through three different block types, which required scrolls of either short, medium, or large extents in 50% of trials. We refer to these trials as *inducer trials*. These trials were expected to affect the direction-dependent component of click position selections. Whether this was the case was scrutinized in *test trials*, which required scrolls of the same extent regardless of block type. They were hence directly comparable. If the above hypothesis is correct, we expect that the initial vertical cursor positions in test trials depends on the eccentricity of inducer trials administered in the same block. That is, we expect that the average demand of a block affects click positions and not only the immediate task demands of the upcoming scroll. In addition, if participants gradually adapt their click positions, the effect of inducer eccentricity should increase within blocks.

### Method

#### Participants

Data of 45 participant were successfully collected in 2021 (37 female, 8 male; 42 right handed, 2 left handed, 1 ambidextrous). Their mean age was 27 years and ranged from 19 to 59 years. They were recruited from the participant pool of the Department of Psychology of the University of Würzburg and hailed predominantly from Würzburg or other German cities. Most participants (n = 35) reported to have used the mouse as instructed, a few used the touchpad (n = 9), and someone used both input devices (n = 1). Participants received a compensation of €5.

To estimate the power for Experiment 2, we computed the means and standard deviations of trials with target numbers 4 and 6 with center start positions of Experiment 1 for each participant. To simulate an individual trial of Experiment 2, we sampled randomly from normal distributions with the respective means and standard deviations for each trial’s target number and participant and added a hypothetical effect of inducer eccentricity, which was assumed to increase linearly within blocks. We simulated 10.000 experiments for sample sizes of 6, 12, …, and 48 participants assuming an effect of either 0.03 units (roughly corresponding to the differences between adjacent target numbers in Experiment 1) or a more conservative 0.01 units between adjacent levels of eccentricity at the end of each block. The power for the expected interaction between target number and eccentricity exceeded 95% for samples sizes of at least 36 and 12 participants for the 0.01 and 0.03 unit effect estimates, respectively. An additional modulation of this interaction within blocks could be detected with a power of 95% only for the 0.03 unit effect estimate (given the probed sample sizes) for sample sizes of at least 30 participants. We hence planned to collect data for at least 36 participants but offered more slots in case of no-shows or recording failures, resulting in a final sample size of 45 participants.

### Apparatus, stimulus, and procedure

Experiment 2 deviated from Experiment 1 as follows (ESM-Movie-2 shows a screen recording). The number line now consisted of 11 vertically arranged squares (0.14 × 0.14 units) numbered from 10 (top) to 20 (bottom). The target squares were blue and resized to match the squares of the number line (0.14 × 0.14 units). The start square (0.1 × 0.1 units) was always vertically centered and positioned 0.29 units to the left or right of the center of the number line.

At the beginning of each trial, the numbers 12 to 18 were fully visible, and the start button was presented on the screen. If participants released the number line within 0.014 units from the target position, light blue sparkles moved around the number line square with the target number on it for 0.5 s. Then, the next trial started.

The experiment was divided into three blocks, each of which contained a different set of target numbers. The targets 10, 13, 17, and 20 were presented in the high inducer eccentricity blocks, the targets 12, 13, 17, and 18 were presented in the medium inducer eccentricity blocks, and the targets 13, 14, 16, and 17 were presented in the low inducer eccentricity blocks. Note that the test target numbers 13 and 17 appeared in all blocks, whereas the remaining inducer targets differed between blocks. Blocks were presented in pseudorandom order. Each block comprised three subblocks, in which each combination of target number and the side of the start square were presented four times in pseudorandom order, resulting in 32 trials per subblock. A self-terminated pause was presented at the end of every subblock. The experiment took approximately 25 min.

## Results

**Fig. 3:**
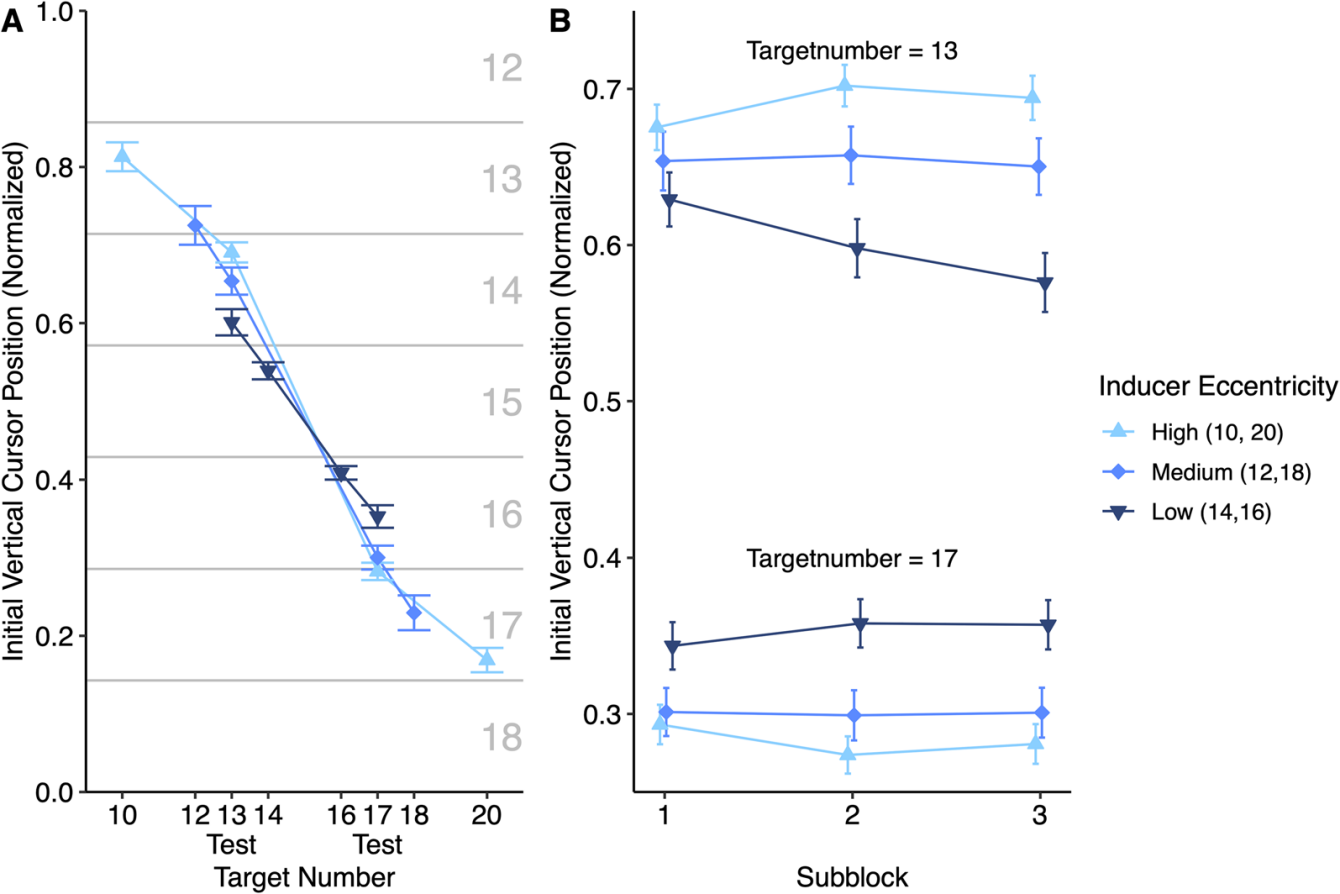
Results of Experiment 2. Note: The figures show the normalized initial vertical cursor position. Values of 0 and 1 refer to the bottom and top edge of the screen, respectively. Error bars show 1 standard error of the mean. **A** The gray lines and numbers reflect the initial position of the number line for reference

As mouse users and touchpad users only differed significantly in how they adapted their behavior within blocks to the experimental conditions, we did not consider this factor in the following analyses (Table ESM-3, Figure ESM-3). Figure 3A shows initial vertical cursor positions for inducer and test trials averaged over subblocks. Descriptively, the figure shows a strong effect of the target number, as in Experiment 1. In addition, it shows that click positions in test trials depend on which types of inducers were presented in the remaining trials. This effect is shown in Fig. 3B, which splits the data by subblock. The averaged initial vertical cursor positions in test trials were entered in a repeated measures ANOVA with factors of target number (13, 17), inducer eccentricity (high, medium, low), and subblock (1, 2, 3), applying the Greenhouse–Geisser correction for sphericity violations.

Click positions depended strongly on the target number, *F*(1,44) = 192.77, *p* < .001, η^2^_*G*_ = .72. Importantly, target number and eccentricity interacted, *F*(1.86,81.96) = 23.95, *p* < .001, η^2^_*G*_ = .09, ε = .93. As expected, the higher the eccentricity of the inducer targets, the further were the click positions from the screen center. Contrasts revealed this interaction when comparing the low and medium eccentricity conditions, *t*(44) = 4.06, *p* < .001, *d*_*z*_ = 0.61, as well as the medium and high eccentricity condition, *t*(44) = 2.29, *p* = .027, *d*_*z*_ = 0.34. Finally, the three-way interaction was significant, *F*(2.68,117.95) = 5.63, *p* = .002, η^2^_*G*_ = .01, ε = .67. Descriptively, the interaction between eccentricity and target number increased from the first to the second subblock of the experiment. This interpretation is supported by a significant contrast comparing the effect of target number and high vs. low eccentricity in the first and second subblock, *t*(44) = 3.35, *p* = .002, *d*_*z*_ = 0.50. A similar contrast for the second and third subblock did not indicate further changes, *t*(44) = 0.41, p = .688, *d*_*z*_ = 0.06.

In addition, there was a numerical tendency for higher eccentricities resulting in minimally higher click positions, *F*(1.91,84.09) = 2.91, *p* = .062, η^2^_*G*_ = .00, ε = .96. Click positions tended to decrease slightly during blocks, *F*(1.86,81.67) = 2.92, *p* = .063, η^2^_*G*_ = .00, ε = .93. Moreover, the effect of eccentricity per se tended to increase over subblocks, *F*(2.93,128.98) = 2.55, *p* = .060, η^2^_*G*_ = .00, ε = .73. There was no significant interaction between target number and subblock, *F*(1.49,65.52) = 1.25, *p* = .284, η^2^_*G*_ = .00, ε = .74.

### Short discussion

Experiment 2 tested whether specific click positions for scrolling actions depended on what other kinds of scrolling actions were required in the same context. This was borne out and the magnitude of the effect was substantial. For example, the difference in click positions for scrolling a low vs. medium eccentricity inducer target to the screen center was only about four times larger than the effect that the mere presence of these inducer trials had on centering otherwise identical test targets. This suggests that the overall range of scrolling actions required in the experimental context has a considerable effect on click position selection in specific trials.

How we execute actions does not only depend on current goals but also on previous choices (Kelso et al., 1994; Rosenbaum & Jorgensen, 1992). This raises the question of whether the effect of inducer eccentricity is mainly the result of transient priming from the previous trials. However, if that was the case, we would not expect an increase of the interaction between inducer eccentricity and target number over subblocks. In addition, we checked whether the number of test trial repetitions affected the interaction (see section “effect of test trial repetitions” in supplemental material). While the interaction tended to be slightly smaller when a test trial was directly preceded by another test trial, a strong interaction persisted irrespective of the recent trial history. This suggests that the effect of eccentricity mostly represents a gradual adaptation to the scrolling actions required in a task context and does not predominantly result from trial-to-trial priming.

### Effect of preceding actions on click position selection in Experiments 1 and 2

Experiment 2 supported our hypothesis that click selection is based on the average requirements of scrolls in different *directions*. In this section, we want to further explore which variables determine the adjustment of the click position to the anticipated *extent* of the scrolling movement. To do so, we harness that features of previous actions are often carried over to subsequent actions (*motor hysteresis*, Kelso et al., 1994; Cohen & Rosenbaum, 2004; Schütz & Schack, 2019; Valyear et al., 2018). That is, by determining which variables of past clicks affect subsequent clicks, we hope to infer the variables that underly click position selections.

According to our interpretation, click positions depend on two factors. First, a click position is selected based on the direction of the scrolling action and the typical requirements of scrolling actions in the current task context. This click position is then further adapted to the absolute extent of the required scrolling action. If that was the case, the absolute extent of the scrolling action and henceforth the resulting eccentricity of the click (i.e., absolute distance from a reference point such as the center of the scrollbar) would underly click position selection and might be a variable that affects subsequent click positions. Consequently, it could be predicted that the more eccentric the click position in the preceding trial was, the more eccentric it will be in the next trial—irrespective of whether the direction of the scrolling action changes.

By contrast, based on experiments on the selection of grasp orientations and positions for object manipulation it has been suggested that actions are planned in terms of arm postures and that previously absolute postures determine action selection (Cohen & Rosenbaum, 2004). For example, participants tend to repeat specific grasp orientations (Kelso et al., 1994; Rosenbaum & Jorgensen, 1992; Schütz & Schack, 2019) or grasp positions (Cohen & Rosenbaum, 2004) when repeatedly grasping objects for manipulation. Applied to the scrolling task, this would imply that the click position selection would be characterized by the absolute position of the previous click. According to this account, higher click positions in one trial should be always followed by higher click positions in the subsequent trial.

To compare both accounts, we predicted initial vertical click positions with linear mixed effect models for both experiments. Trials were included in the analysis if they and their immediate predecessors were finished within 10 s and if they were not the first trial in a block (98% and 96% of all trials for Experiments 1 and 2, respectively). According to the first account, we included the eccentricity of the previous trial (absolute distance of the initial vertical click position from the screen center), centered the range of possible values on zero, and multiplied it by 1.0 or − 1.0, depending on whether the current trial required an upward or downward scroll. For example, a click on the top of the screen in trial n − 1 resulted in a value of 0.25 for this predictor if trial n involved a downward scroll and − 0.25 if it required an upward scroll. According to the latter account, we also included the initial vertical cursor position of the previous trial.

In addition to these predictors of interest, we considered the signed amplitude of the scrolling action in the current trial and the direction of the scrolling action to account for additional effects of the direction that are not captured by the amplitude per se (coded as − 0.5 vs 0.5 for down and up). We included the vertical start position (− 0.4, 0, 0.4) for Experiment 1 only. The factor inducer eccentricity (0.14, 0.43, 0.71, distance of inducer to screen center) was only considered for Experiment 2. Note that all predictors (except scroll direction) are coded in units relative to the screen height, allowing to directly compare their influence on click positions.

We used R (R Core Team, 2022), *lme4* (Bates et al., 2015) and *afex* (Singmann et al., 2023) to fit a linear mixed effect model with the above predictors as fixed effects and by-participant random slopes for all predictors. The marginal and conditional R^2^ were .84 and .88, respectively, for Experiment 1 and .77 and .87, respectively, for Experiment 2. Table 1 summarizes the fixed effects. P values were derived with F tests using Satterthwaite’s (1941) approximation of degrees of freedom. All included predictors had low VIF values (≤ 3.01) indicating low multicollinearity. Additionally, weights of previous click position and previous click eccentricity did not change substantially (i.e., by less than 0.01) when the respective other predictor was not included in the model. Excluding the respective other predictor had no effect on statistical significance. Not surprisingly, the initial vertical click position was inversely related to the signed amplitude. That is, upward scrolls resulted in lower clicks and vice versa. In addition, the scrolling direction had an additional effect. However, as scrolling direction and signed amplitude of the current trial are correlated predictors, their weights should not be interpreted. Note, that the estimated weights of the fixed effects are very similar between experiments. The start position (Exp. 1) and the inducer eccentricity (Exp. 2) had a smaller but significant effect on click positions.

**Table 1:** Fixed effect statistics of linear mixed effects models for Experiments 1 and 2

Fixed effect	Exp. 1				Exp. 2			
	$\hat{\beta }$	SE	*F*	*p*	$\hat{\beta }$	SE	*F*	*p*
Scr. amplitude	− 0.31	0.02	*F*(1,36.01) = 394.95	< . 001	− 0.33	0.02	*F*(1,45.15) = 321.65	< .001
Scr. direction	− 0.22	0.02	*F*(1,35.84) = 170.05	< . 001	− 0.18	0.02	*F*(1,44.32) = 134.84	< .001
Start position	0.05	0.01	*F*(1,36.01) = 59.42	< . 001				
Inducer eccentricity					0.02	0.01	*F*(1,44.84) = 8.86	.005
Prev. click position	0.01	0.01	*F*(1,26.61) = 3.39	.077	0.01	0.00	*F*(1,31.30) = 2.62	.116
Prev. click eccentricity	0.14	0.02	*F*(1,35.12) = 69.22	< . 001	0.40	0.03	*F*(1,44.17) = 206.24	< .001

In line with our hypothesis, the eccentricity of the previous initial vertical cursor position (combined with the current scrolling direction) had a considerable effect in both experiments. By contrast, the position of the initial vertical click position in the previous trial did not contribute significantly to the model for either experiment. By contrast, the weights of eccentricity were about an order of magnitude larger than the estimates for the absolute previous position.

To summarize, the analyses revealed two important facts. First, click position selections depend partially on previous click position selections. Second, not all facets of an action inform the next one to the same extent. In both experiments, the feature of the previous trial that had the largest effect on click position was the eccentricity of the click. This suggests that the eccentricity of the click position and not its absolute position is a crucial parameter of click position selection.

## Discussion

Scrolling actions are commonplace in digital environments. We addressed whether and how click positions on screen objects depend on the upcoming scrolling action. Experiment 1 revealed a tight relationship between click position and the intended scrolling action. While click position selection certainly facilitated scrolling without having to release and re-click, click positions selections did not strictly optimize any obvious criterion. Based on observations from object manipulation tasks, we suggested that click positions are heuristically selected by a two-component process. First, click positions are selected that allow easy scrolling in the required direction in the current task context. Second, the selected click positions are adapted to the extent of the planned scroll. The first aspect of the hypothesis was further tested in Experiment 2, which revealed that the scrolling amplitudes in the current task context affected click position selections for individual trials. The second aspect found support by analyzing the effect of previous trials on click position selections in subsequent trials for both experiments. It was revealed that the eccentricity but not the absolute previous click position affected click positions selection in the subsequent trial. In the following, we want to discuss the determinants of anticipatory actions, their relationship to anticipatory action in real-world object manipulation tasks, and potential for application.

### Determinants of click position selections

Click positions were attuned to the direction and extend of the upcoming scrolling action. However, click positions were also affected by past actions. First, Experiment 2 revealed that the overall task context affects click positions. For example, if participants were working on a block that frequently required extended scrolls and hence eccentric click position, they also selected more eccentric click positions for shorter scrolls. This influence got stronger the more often participants had sampled experience from that context. As with every blockwise manipulation, it is difficult to distinguish whether this influence is determined reactively, by memory retrieval of previous scroll encounters, or proactively, by the expectation that forthcoming trials would obey to the same context (Schmidts et al., 2020). On top of this sustained effect, click positions depended on a more transient influence of the previous trial’s click eccentricity. Both effects had a substantial impact on click positions.

On the other hand, factors that could be expected to affect click positions had only a relatively small effect—if any. First, while the position of the cursor at the beginning of a trial affected click positions consistently, the magnitude of the effect was relatively small. Second, whether people scrolled with the touchpad or the mouse had only a minor effect in our experiments. While the lack of significant differences can be most likely attributed to insufficient power, any differences between both modes of interaction were also numerically small.

In our analysis, we focused on the average behavior. This raises the questions to which extent behavior differed between participants and whether participants employed different strategies. Indeed, participants varied in how strongly they adapt the click position to the scroll and to some extent also with respect to the shape of this relationship (ESM-Figure-2, ESM-Figure-4). However, participants apparently do not fall into clearly definable subgroups that rely on qualitatively different strategies (ESM-Figure-6). The variability among participants does not relate significantly to age or gender (ESM Table-5, ESM Table-6). While the input device of course affects click position selections (ESM Table-7), the behaviors of mouse and touchpad users also overlapped considerably. Finally, we found that participants that initiated the movement toward the scrollbar more slowly show a more nonlinear relationship between target number and click position and completed the scrolling action more slowly (ESM Table-8). However, this correlation seems to be indicative of overall performance rather than the result of a focus on either quick movement initiation or more efficient movements. In conclusion, differences between participants appeared to be a matter of degree but not of kind.

In summary, we suggest that the present behavior can be best understood as a mix of motor-related and cognitive factors. We would argue that click position selection is generally shaped by the preference to complete scrolling actions without having to release and re-click the scrolled object—which reduced the time needed as well as the overall movements required. The general relationship between the upcoming scrolling action and click position selection as well as the effect of inducer eccentricity in Experiment 2 are in line with this assumption. However, within this constraint, it can hardly be argued that click position selections are strictly optimized. This becomes evident by the relatively large effect of variables that are unrelated to the task at hand (e.g., previous trials) and the small effect of arguably relevant variables (e.g., start position). Rather, we suggest that participants employ a simplified, heuristic mode of planning, which coarsely approximates optimal behavior. That is, the costs of slightly suboptimal movements might be outweighed by the benefits of easier click position planning (Cohen & Rosenbaum, 2004; Schütz & Schack, 2019; Valyear et al., 2018). However, interestingly this does not mean that participants always rely on the simplest mode of planning possible. In the low eccentricity condition of Experiment 2, all scrolls could be accomplished without re-clicking by clicking the bar on the central square, which was also closest to the start squares. Nevertheless, only a minority of participants used this strategy. This suggests that participants have a strong tendency to consider upcoming actions during action selection (c.f., Herbort & Butz, 2010). How strongly this affects participants may relate to individual factors as well as the work environment.

### Anticipatory action in real and virtual environments

Anticipatory actions have been extensively studied in object manipulation tasks (Rosenbaum et al., 2012), but also in virtual reality environments (Herbort & Kunde, 2019), touch screen interactions (e.g., Olafsdottir et al., 2014), or other scenarios (Grießbach et al., 2021). In those cases, behavior is thought to be primarily adapted to reduce the impact of the limits of the human motor system. By contrast, in our task the limiting factor was assumed to be primarily the screen border, which prevented cursor movements and thus scrolls of indefinite amplitude. Nevertheless, there were several similarities between behavior in the present task and real-world object manipulations. First, our experiments revealed a nonlinear relationship between click positions and the required scrolling action, which closely (and surprisingly) resembles the effect of upcoming rotations on grasp orientation (Herbort, 2013; Herbort & Butz, 2012). Second, effects of previous trials are common in grasp selection for object manipulation (Cohen & Rosenbaum, 2004) and were also consistently revealed in the present experiments. Likewise, as in our Experiment 1, very small effects of the initial posture on grasp selections for object manipulation have been reported (Herbort & Butz, 2012). In summary, there appears to be a commonality between both tasks which suggests that findings from physical object manipulation tasks may be applied to virtual settings and vice versa with some caution.

### Limitations

As our experiments were conducted online, we had no control over participants’ computer configurations, including factors such as the specific mouse models or the mouse setting (e.g., mouse sensitivity). Moreover, some participants chose to use the touch pad and not the mouse as instructed. On the one side, this variability may have limited the potential to detect smaller effects. On the other side, despite this variability, the intended scroll was inversely related to the initial vertical click positions in each individual participant, at least numerically. Moreover, differences between mouse and touchpad users were systematic but comparatively small (see also ESM-Figure-1, 3, and 6). This suggests that the specifics of the input device had only a minor effect on click position selections. By extension, it can be speculated that also variability within both classes of input devices, for example, due to the different mouse models or settings, may have little effect on the general data pattern. By contrast, the reported relationship between scrolling action and click position appears to be robust over a wide range of computer setups.

Another limitation concerns the constraints on cursor movements. A priori, we considered the screen borders as the primary constraint for cursor movements, which necessitated to adapt click positions to the scrolling task. However, cursor movements may have been subject to further constraints. For example, mouse users may have reached the limits of their mouse pads or desks in some trials. Likewise, touchpad users may have reached the borders of their touch pads. Just as the screen border, these constraints restricted cursor movements to a limited area and thus imposed similar limitations. Moreover, as mouse and touch pad users click position selections differed only little, we suspect that such additional constraints had a relatively small effect on our results.

Finally, our experiment required moving a screen object to a specific position with relatively high accuracy requirements. Typical everyday examples include positioning larger objects when preparing a slide show, selecting a specific view on a map application, or using a picker. However, in other scrolling tasks the accuracy requirements might be much looser, or the amplitude of the scrolling actions might be even uncertain, for example, when scrolling in search for a specific contact on a phone or a file in a folder. In the latter case, participants still adapt click positions strongly to the scrolling direction (Zhao et al., 2014), suggesting that anticipatory click position selections as observed in our task are ubiquitous although our task only reflects a subset of the scrolling task that users typically encounter.

### Implications for applications

In large-dataset computer application, only a fraction of the data that could be accessed by the user is prepared for display. For example, in map applications, only information for the current viewport and zoom level may be loaded. To allow seamless interactions, algorithms have been devised that predict what data the user is going to access in the immediate future (e.g., Battle et al., 2016; Doshi et al., 2003). Our experiments have shown that click positions can be an excellent predictor for upcoming scrolling actions. Incorporating this information might allow more efficient data prefetching and thus smoother interactions.

Fitts’ law (1954) has proven to be a successful tool for understanding and modeling human–computer interaction (MacKenzie et al., 1991; Zhao et al., 2014, 2015), because it describes a strong invariance of human behavior. Our data showed that not only the characteristics of an immediate cursor target determines user behavior but also the intended interaction. Thus, anticipatory effects should be considered in HCI models. Moreover, our data have direct implications for the application of Fitts’ law in human–computer studies. According to Fitts’ law, one way to improve interactions is to increase the area of to-be-clicked screen objects. Our data suggest that users who intent to move a screen object—as in scrolling or dragging—may not aim for the object center but to positions closer to the edge of the object. This increases accuracy requirements and might even decouple them to some degree from the objective object dimensions. Experiment 2 suggests that this may even be the case if the intended object manipulation might be accomplished without adapting the click position at all. In user interaction studies, such anticipatory tendencies should be kept in mind when considering the effect of object dimensions (as in Fitts’ law) on interaction performance. When designing user interfaces, methods should be considered to nudge users to aim for the object center if the adaptation of the click position to the intended object displacement is likely to be unnecessary (e.g., by displaying a salient handle) (Additional files: 1, 2, 3).

## Summary

We examined how participants select click positions for scrolling actions. Participants adapted click positions strongly to the intended scrolling direction. We suggest that click position selection is based on a heuristic process that is primarily determined by the direction of the intended scroll and the requirements of past interactions. Moreover, click positions were adapted to intended scrolls even when this was not strictly necessary. We conclude that people have a strong tendency to consider subsequent actions when interacting with screen objects and that considering this factor might be used to improve human–computer interaction and our understanding thereof.

### Supplementary Information

**Additional file 1.** Electronic Supplemental material, containing additional analyses, plots, and tables. It is referenced as Table ESM-X or Figure ESM-X.

**Additional file 2.** ESM-Movie-1.

**Additional file 3.** ESM-Movie-2.

## Data Availability

The datasets generated and/or analyzed during the current study are available in the OSF repository, https://osf.io/h3nqk/?view_only=1ed6718b29544ac89af11155b34807ce.

## Footnotes

1 Targets 1 and 9 could only be centered in one go when clicking on the single highest or lowest visible square, respectively. All clicks that allowed centering the target without release and re-click were necessarily shortest possible movements.
