# Supplementary material for: Where scrollbars are clicked, and why
Source: Cogn Res Princ Implic. 2024 Apr 19;9:23. doi: 10.1186/s41235-024-00551-z (PMC11026321; doi:10.1186/s41235-024-00551-z)
Supplement: Supplementary file 1 — Additional file 1. Electronic Supplemental material, containing additional analyses, plots, and tables. It is referenced as Table ESM-X or Figure ESM-X. [file 41235_2024_551_MOESM1_ESM.pdf]

**Supplemental Material to:**

**Where Scrollbars are clicked, and why?**

Oliver Herbort, Philipp Raßbach, and Wilfried Kunde
Department of Psychology, University of Würzburg, Würzburg, Germany

## Experiment 1

### Effect of Horizontal Start Position

Table ESM-1 shows the results of an ANOVA on the initial vertical cursor position with within-participant factors of target number (1, 2, 3, 4, 6, 7, 8, 9), vertical start position (low, center, high), and horizontal start position (left, right).

**Table ESM-1: ANOVA on initial vertical cursor position with factors of target number, vertical, and horizontal start position for Exp. 1**

| Factor | *F* | *p* | *ges* | $\varepsilon_{GG}$ |
|---|---|---|---|---|
| Target number | $F(1.48,51.91) = 698.93$ | < .001 | .93 | .21 |
| Vertical start pos. | $F(1.29,45.06) = 50.85$ | < .001 | .04 | .64 |
| Horizontal start pos. | $F(1.00,35.00) = 1.39$ | .246 | .00 | - |
| Target number x vertical start pos. | $F(8.26,289.26) = 1.59$ | .124 | .00 | .59 |
| Target number x horizontal start pos. | $F(5.38,188.13) = 0.25$ | .949 | .00 | .77 |
| Vertical start pos. x horizontal start pos. | $F(1.94,67.99) = 1.47$ | .238 | .00 | .97 |
| Target number x vertical start pos. x horizontal start pos. | $F(9.04,316.42) = 0.76$ | .658 | .00 | .65 |

*Significant effects are marked green.*

### Effect of Input Device

Table ESM-2 shows the results of an ANOVA on the initial vertical cursor position with within-participant factors of target number (1, 2, 3, 4, 6, 7, 8, 9), vertical start position (low, center, high), and between-participant factor input device (touchpad, mouse). The participants (n = 3) who responded to have used both input devices were not included in the ANOVA. The effect of the input device manifested in the three-way interaction. Descriptively, touchpad users clicked closer to the screen center than mouse users.

**Table ESM-2: ANOVA on initial vertical cursor position with factors of input device, target number, and vertical start position for Exp. 1**

| Factor | *F* | *p* | *ges* | $\varepsilon_{GG}$ |
|---|---|---|---|---|
| Input device | $F(1.00,31.00) = 0.53$ | .471 | .00 | - |
| Target number | $F(1.52,47.23) = 688.64$ | < .001 | .94 | .22 |
| Vertical start pos. | $F(1.28,39.80) = 46.17$ | < .001 | .06 | .64 |
| Input device x target number | $F(1.52,47.23) = 1.08$ | .331 | .03 | .22 |
| Input device x vertical start pos. | $F(1.28,39.80) = 2.30$ | .132 | .00 | .64 |
| Target number x vertical start pos. | $F(8.96,277.88) = 1.36$ | .205 | .00 | .64 |
| Input device x target number x vertical start pos. | $F(8.96,277.88) = 2.93$ | .002 | .01 | .64 |

*Significant effects are marked green.*

**Figure ESM-1**

*Initial vertical cursor position by input device, target number, and start position in Exp. 1*

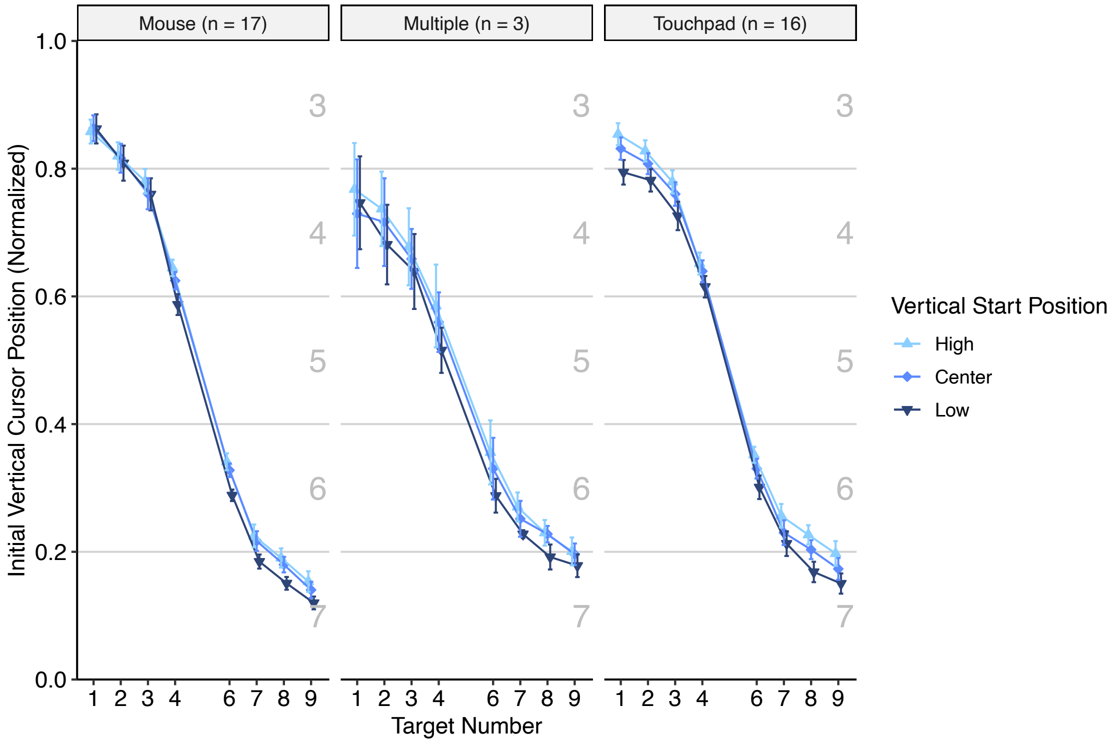


Note: Error bars show 1 SEM.

**Data of individual participants**

Figure ESM-2 shows the initial vertical cursor position of individual participants. All participants show a strong relationship between target number and initial vertical cursor position. If participants always clicked on the target number, behavior would resemble those of participants Mouse-03 and Mouse-04. If participants tried to end their movements at the screen border, one would expect lower click positions (ca 0.2) for target number 4 than for target number 6 (ca 0.8). No participant showed this pattern.

**Figure ESM-2**

*Initial vertical cursor positions by target number, start position, and participant for Exp. 1*

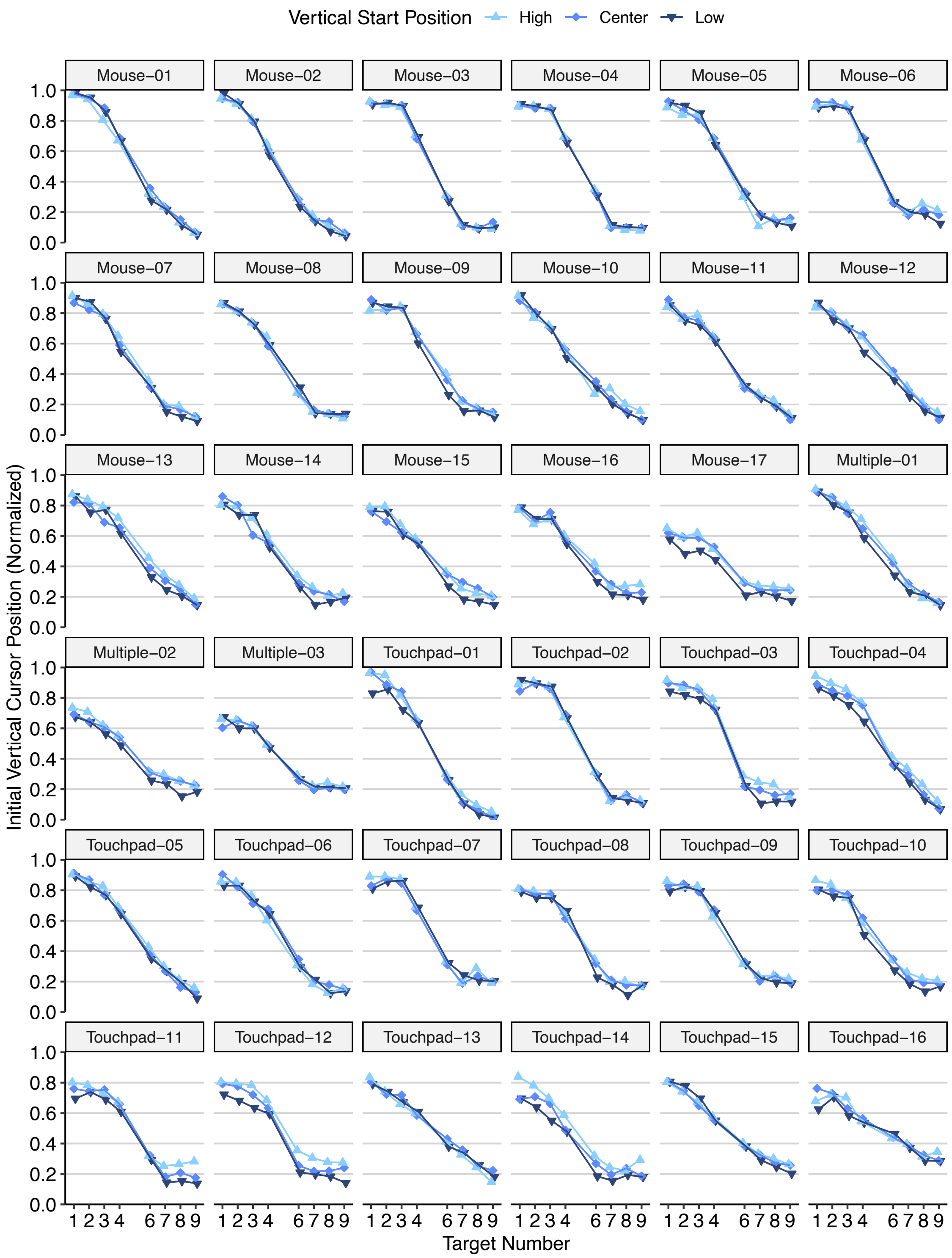


*Note: Participants have been sorted by input device and the effect of target number on initial vertical click position.*

# Experiment 2

## Effect of Input Device

Table ESM-3 shows the results of an ANOVA on the initial vertical cursor position with within-participant factors of target number (13, 17), inducer eccentricity (low, medium, high), and between-participant factor input device (touchpad, mouse). The participant (n = 1) who responded to have used both input devices was not included in the ANOVA. The effect of the input device manifested in the four-way interaction. Figure ESM-3 shows that the interaction of target number and eccentricity increased over subblocks for mouse users but not for touchpad users.

**Table ESM-3: ANOVA on initial vertical cursor position with factors of input device, target number, inducer eccentricity, and subblock for Exp. 2**

| Factor | *F* | *p* | *ges* | $\varepsilon_{GG}$ |
|---|---|---|---|---|
| Input device | $F(1.00,42.00) = 0.23$ | .631 | .00 | - |
| Target number | $F(1.00,42.00) = 136.32$ | < .001 | .65 | - |
| Inducer eccentricity | $F(1.92,80.79) = 1.92$ | .154 | .00 | .96 |
| Subblock | $F(1.86,78.13) = 2.00$ | .146 | .00 | .93 |
| Input device x target number | $F(1.00,42.00) = 0.09$ | .769 | .00 | - |
| Input device x inducer eccentricity | $F(1.92,80.79) = 0.11$ | .890 | .00 | .96 |
| Input device x subblock | $F(1.86,78.13) = 0.04$ | .948 | .00 | .93 |
| Target number x inducer eccentricity | $F(1.87,78.53) = 16.29$ | < .001 | .07 | .93 |
| Target number x subblock | $F(1.48,62.22) = 0.04$ | .916 | .00 | .74 |
| Inducer eccentricity x subblock | $F(2.91,122.25) = 1.85$ | .144 | .00 | .73 |
| Input device x Target number x inducer eccentricity | $F(1.87,78.53) = 0.60$ | .541 | .00 | .93 |
| Input device x target number x subblock | $F(1.48,62.22) = 2.29$ | .123 | .00 | .74 |
| Input device x inducer eccentricity x subblock | $F(2.91,122.25) = 0.31$ | .810 | .00 | .73 |
| Target number x inducer eccentricity x subblock | $F(2.88,120.87) = 0.82$ | .482 | .00 | .72 |
| Input device x target number x inducer eccentricity x subblock | $F(2.88,120.87) = 4.97$ | < .001 | .01 | .72 |

*Significant effects are marked green.*

**Figure ESM-3**

*Initial vertical cursor position by input device, target number, inducer eccentricity and subblock in Exp. 2*

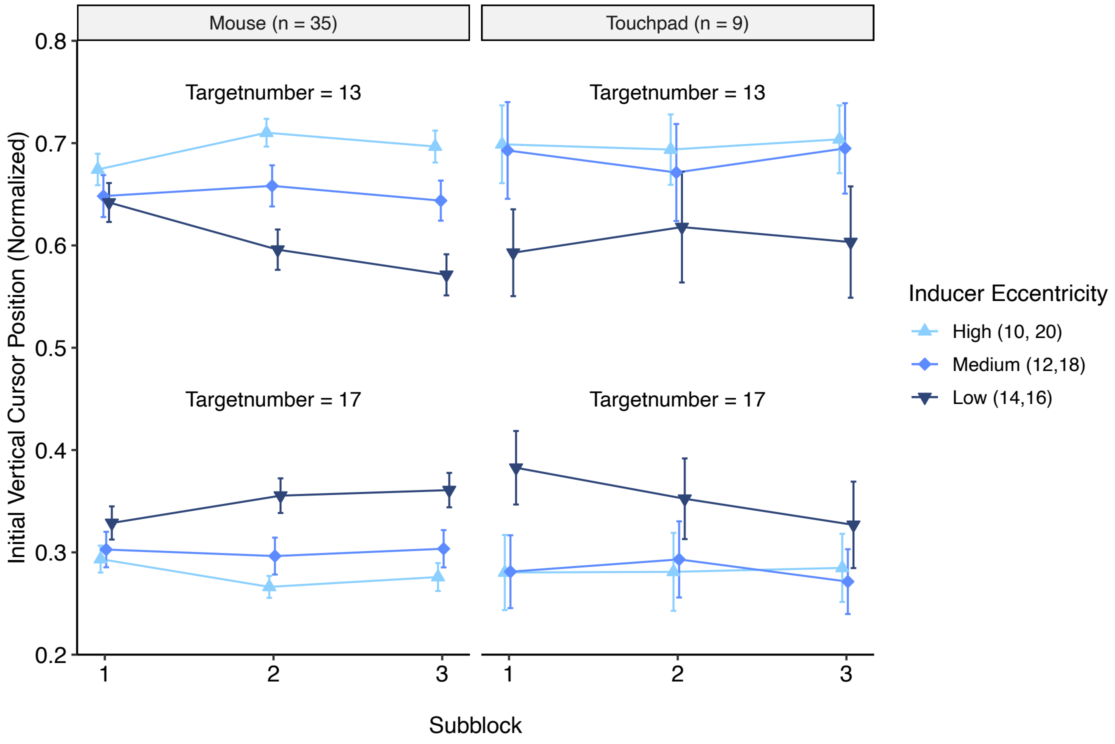


*Note: Error bars show 1 SEM.*

## Data of individual participants

Figure ESM-4 shows the initial vertical cursor position of individual participants.

**Figure ESM-4**

*Initial vertical cursor position in test trials by target number, inducer eccentricity, and participant for Exp. 2*

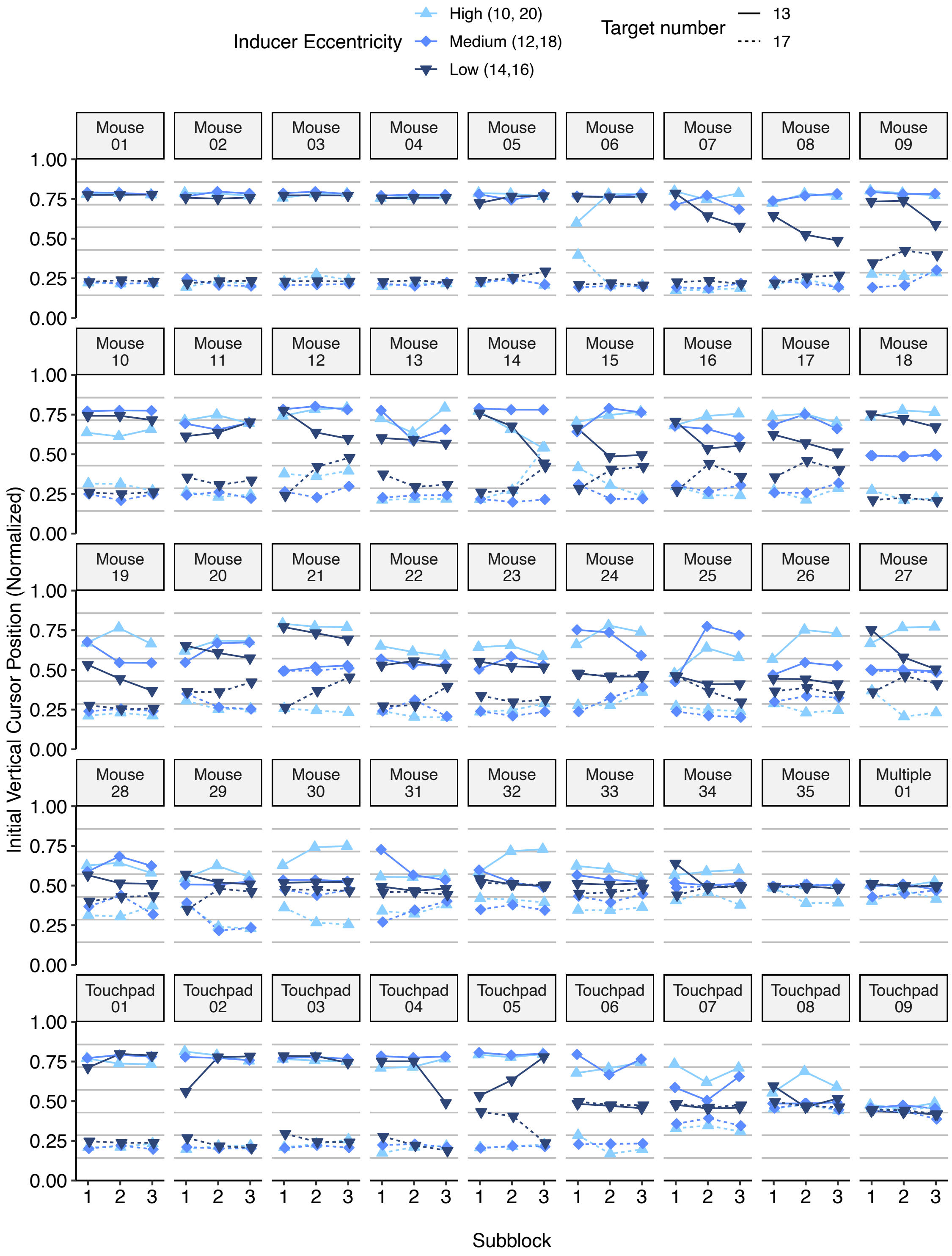


*Note: Participants have been sorted by input device and the effect of target number on initial vertical click position.*

**Effect of test trial repetitions**

To test whether the length of a sequence of test trials affects initial vertical cursor positions, we split the test trial data of Experiment 2 by participant, target number (13, 17), inducer eccentricity (low, high), and the numbers of directly preceding test trials (i.e. repetition: 0, 1, 2 or more). Table ESM-4 shows the results of the ANOVA on the 44 participants that delivered data for all cells. As the critical three-way interaction almost reached significance, we computed contrasts for this interaction for 0 versus 1 repetition and 1 versus 2 or more repetitions. Test trials were more similar when preceded by a test trial than when not, $t(1,43) = 2.60$, $p = .013$, $d_z = 0.39$. However, this effect did not increase when comparing test trials preceded by one versus two or more test trials, $t(1,43) = 0.16$, $p = .874$, $d_z = 0.02$. Finally, ANOVAs with factors of target number and inducer eccentricity conducted separately for each level of repetition all revealed significant interactions, all $F(1,43) \geq 29.45$ , all *p*s ≤ .001, all *ges* ≥ .11.

**ESM Table-4: ANOVA on initial vertical cursor position with factors of target number, inducer eccentricity, and repetition for Exp. 2**

| Factor | *F* | *p* | *ges* | $\varepsilon_{GG}$ |
|---|---|---|---|---|
| Target number | $F(1.00,43.00) = 178.09$ | < .001 | .74 | - |
| Inducer eccentricity | $F(1.00,43.00) = 3.20$ | .081 | .00 | - |
| Repetition | $F(1.88,80.82) = 1.01$ | .364 | .00 | .94 |
| Target number x inducer eccentricity | $F(1.00,43.00) = 52.16$ | < .001 | .13 | - |
| Target number x Repetition | $F(2.00,85.85) = 1.11$ | .333 | .00 | 1.00 |
| Inducer eccentricity x repetition | $F(1.70,73.01) = 1.09$ | .334 | .00 | .85 |
| Target number x inducer eccentricity x repetition | $F(1.73,74.51) = 3.25$ | .051 | .00 | .87 |

*Significant effects are marked green.*

**Figure ESM-5**

*Initial vertical cursor position by target number, eccentricity, and repetition in Exp. 1*

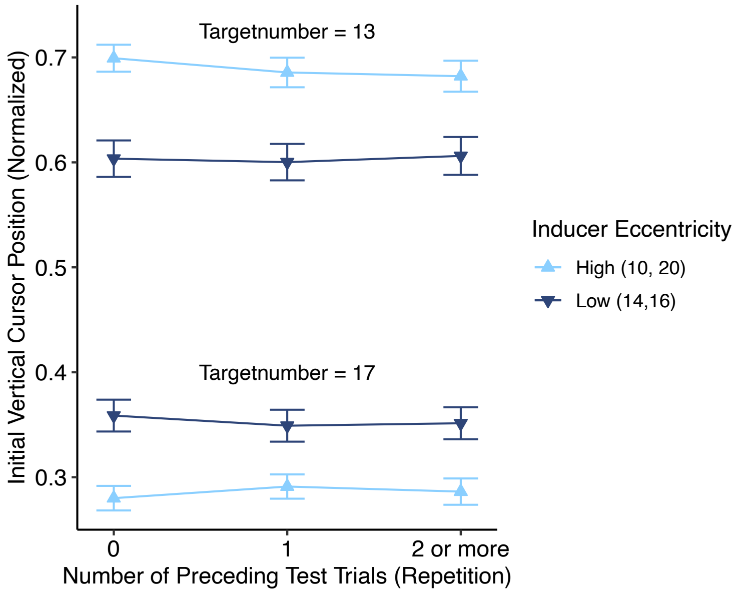


*Note: Error bars show 1 SEM.*

## Interindividual differences

In the following, we consider whether participants can be split into subgroups that apply different strategies. To simplify the analysis and allow the comparison between experiments, we fitted functions to the initial vertical click positions and compare the resulting parameters. More specifically, we first simplified the data. First, we removed general vertical biases by centering the click positions of each participant on zero. Then, the sign of click positions for downward scrolls was reversed. Next, we computed linear regressions for each participant of both experiments (independent variable: absolute extent of the required scroll in screen coordinate; dependent variable: (mirrored) click position. The slope of the linear regression indicates how strongly the extent of the scroll affects click positions. The intercept the effect of the direction per se. We refer to both parameters as *effect of extend* and *effect of direction*, respectively. As an example, consider panel D of Figure ESM-6. The red line shows the model, and the dots represent the participant's mean click positions. The effect of extent corresponds to the slopes of both lines. The effect of direction reflects 50% of the vertical offset between both lines.

Figure ESM-6A shows scatter plots for the effect of direction and extent. Figure ESM-6B shows data of exemplary participants to give an impression of the meaning of the parameters. The figures also shows that the linear model fits the data. The inspection of the figure leads to several conclusions. First, the effect of extent assumes positive values smaller than one. This reflects that increasing the extent of a required scroll by some value alters the initial vertical click positions only by a fraction of that value. Second, the typically positive effect of direction indicates that the movement direction per se appears to have an additional effect on click position. Third, participants differ but cannot be divided into obvious subgroups. An exception may be the five or six participants in the lower left quadrant of Experiment 2, who only slightly, if at all, adapted the click position to the task. Third, while the input device may have a statistical influence on participants behavior, the scatterplots show considerable overlap between mouse users and touchpad users. There is a negative correlation of the effects of direction and extent, $r = .40$, $t(79) = -3.85$, $p < .001$. This reflects that due to the limits of the

screen size, both effects cannot play out in full at the same time. In summary, the data show that participants differ in how they adapt click positions to an upcoming task. However, these differences do not appear to be the result of qualitatively different strategies but rather appear to reflect a continuum.

**Figure ESM-6**

*Parameters of models fitted to each participant's initial vertical click positions.*

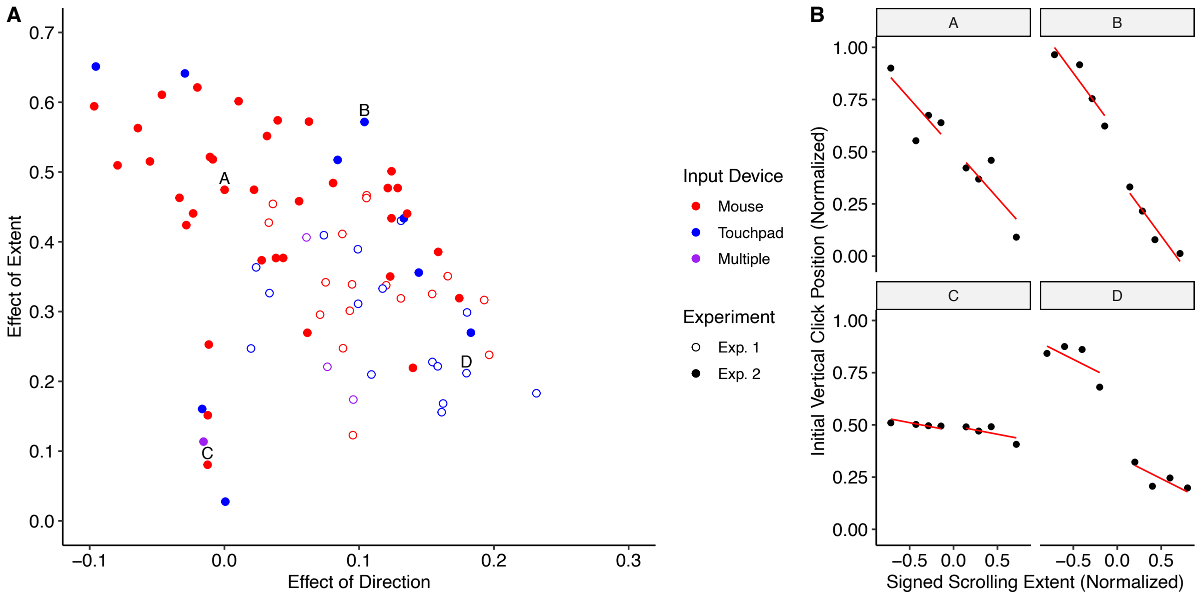


*Note: The data of the marked participants in A are shown in more detail in B. In B, the black dots reflect empirical data whereas the red lines reflect the fit of the model. The effect of extent corresponds to the slope of the red lines. The effect of directions corresponds to 50% of the vertical offset between the lines for upward and downward scrolls.*

In addition, we checked whether click position selections, the average time needed to complete the scrolling action (*scroll time*: time from the first click on the scroll bar to the end of the trial), and the average number of submovements, depended on the time participants took to initiate the movement toward the scroll bar (*approach reaction time:* time from click on start square until the cursor was displaced at least five pixel from the position it had at the time of the click), the participant's age, gender, or input device. The effect of continuous variables was analyzed with correlations. The effect of discrete variables was analyzed with Welsh tests. ESM Tables ESM-5 to ESM-8 show the results. The data reveal that participants that initiate the movement toward the number line slower select click positions stronger on movement direction per se. These participants are also slower in completing the scrolling action, suggesting an overall lower performance, rather than a speed-accuracy trade-off concerning the click position selection. Neither age nor sex predicts click selections, the duration of the scrolling action, or the number of submovements. Finally, as revealed in previous analysis, mouse user's click positions depend more linearly and stronger on the target number (see also Figure ESM-1) and they complete the task considerably faster.

**ESM Table-5: Correlations of age with click position selection and performance**

| Variable | *r* | *t*(79) | *p* |
|---|---|---|---|
| Effect of extent | .03 | 0.23 | .817 |
| Effect of direction | .17 | 1.52 | .132 |
| Scroll time | .08 | 0.73 | .470 |
| Submovements | -.18 | -1.66 | .101 |

**ESM Table-6: Effect of gender (female - male) on click position selection and performance**

| Variable | *t* | *p* | *Cohen's d* | *Difference* |
|---|---|---|---|---|
| Effect of extent | t(43.79) = 0.04 | .966 | 0.01 | 0.00 |
| Effect of direction | t(47.24) = 0.16 | .872 | 0.04 | 0.00 |
| Scroll time | t(42.82) = 1.54 | .131 | 0.38 | 96 ms |
| Submovements | t(33.68) = -0.93 | .358 | -0.24 | -0.05 |

**ESM Table-7: Effect of input device (mouse - touchpad) on click position selection and performance**

| Variable | *t* | *p* | *Cohen's d* | *Difference* |
|---|---|---|---|---|
| Effect of extent | t(39.77) = 2.32 | .025 | 0.59 | 0.08 |
| Effect of direction | t(44.73) = -2.07 | .045 | -0.51 | -0.04 |
| Scroll time | t(49.36) = -6.34 | < .001 | -1.53 | -319 ms |
| Submovements | t(63.45) = -0.18 | .860 | -0.04 | -0.01 |

**ESM Table-8: Correlations of approach RT with click position selection and performance**

| Variable | *r* | *t*(79) | *p* |
|---|---|---|---|
| Effect of extent | .06 | 0.50 | .616 |
| Effect of direction | .36 | 3.45 | < .001 |
| Scroll time | .56 | 6.00 | < .001 |
| Submovements | -.20 | -1.83 | .071 |

*Note:* 123 trials were removed in which the approach RT could not be determined – most likely because the input device allowed placing the cursor directly on the scrollbar without moving it there continuously.
